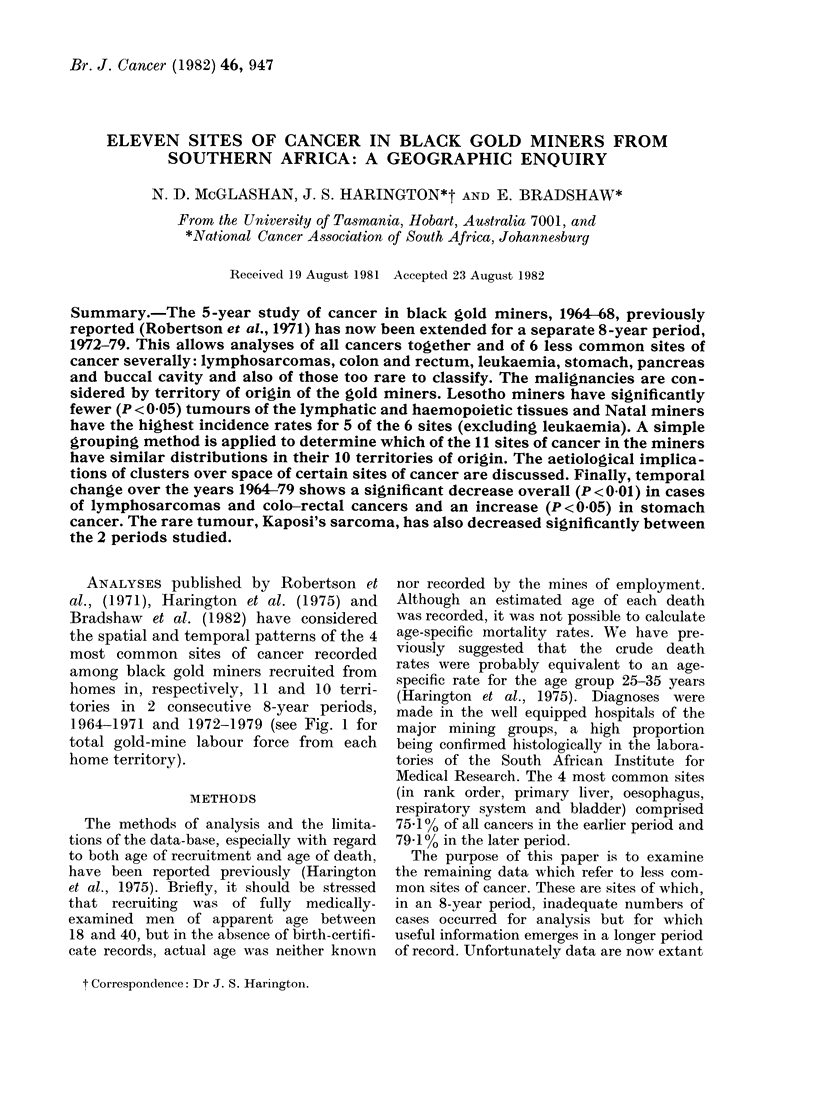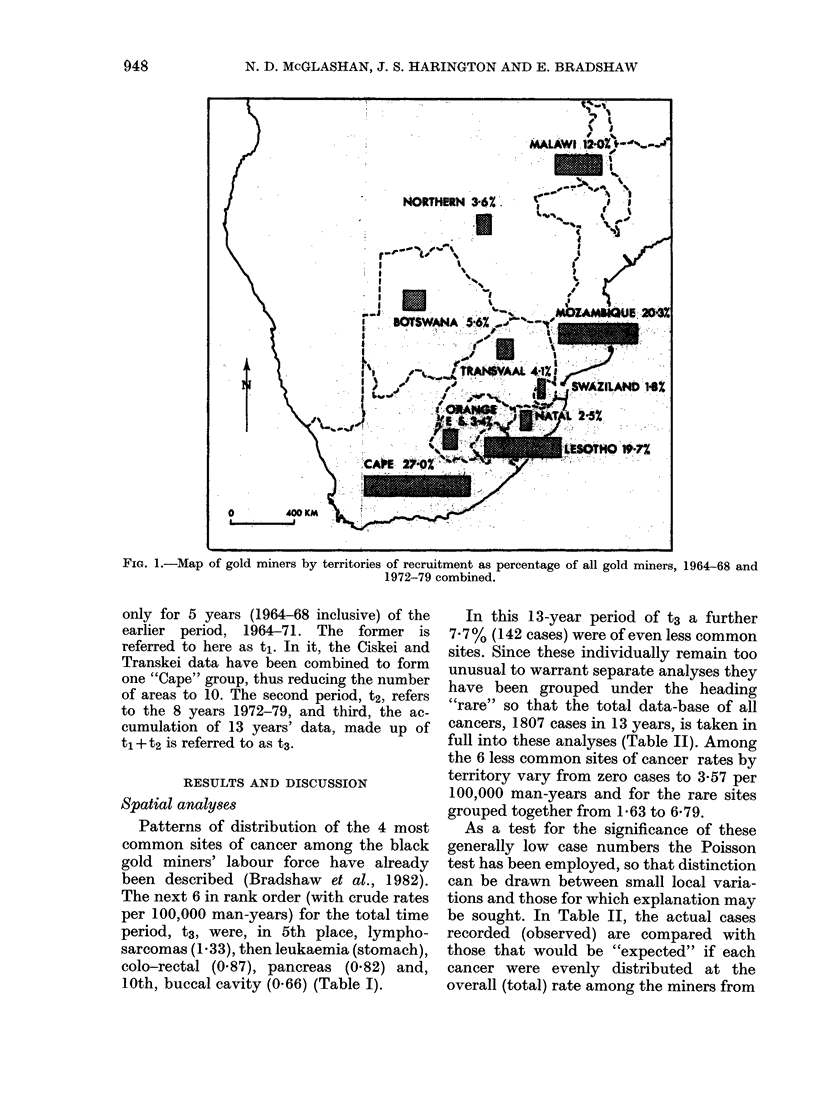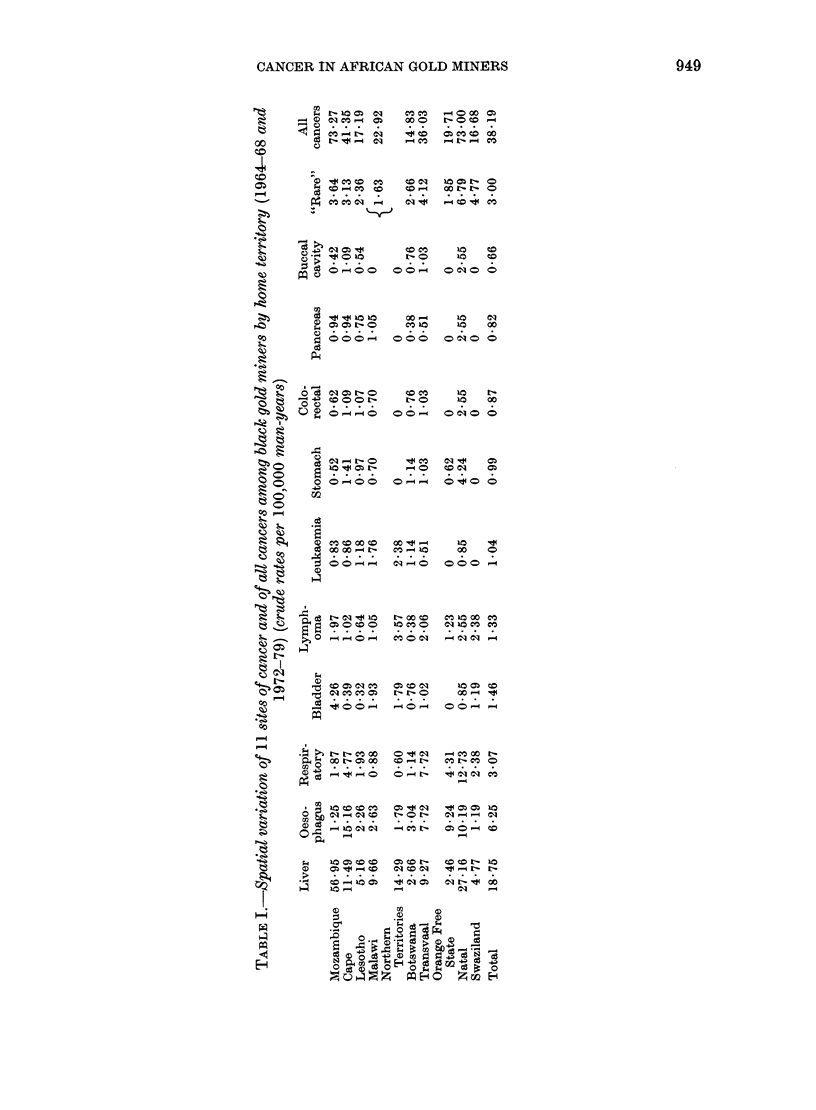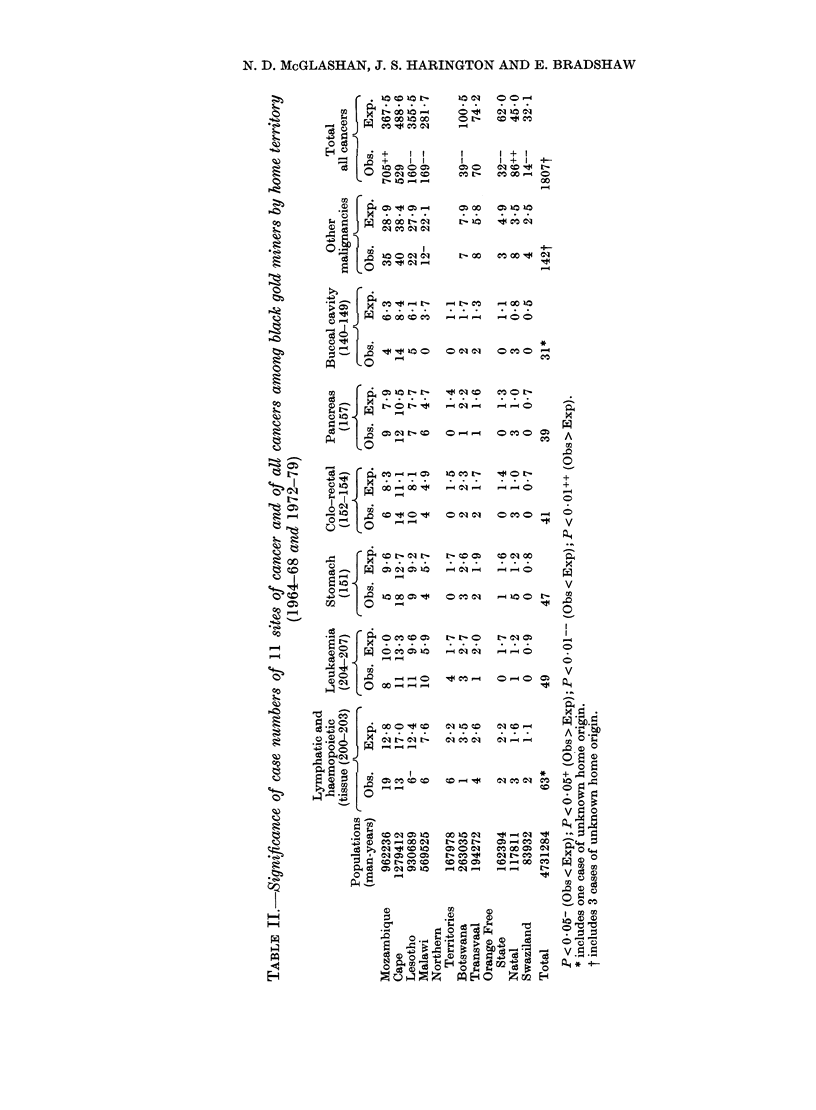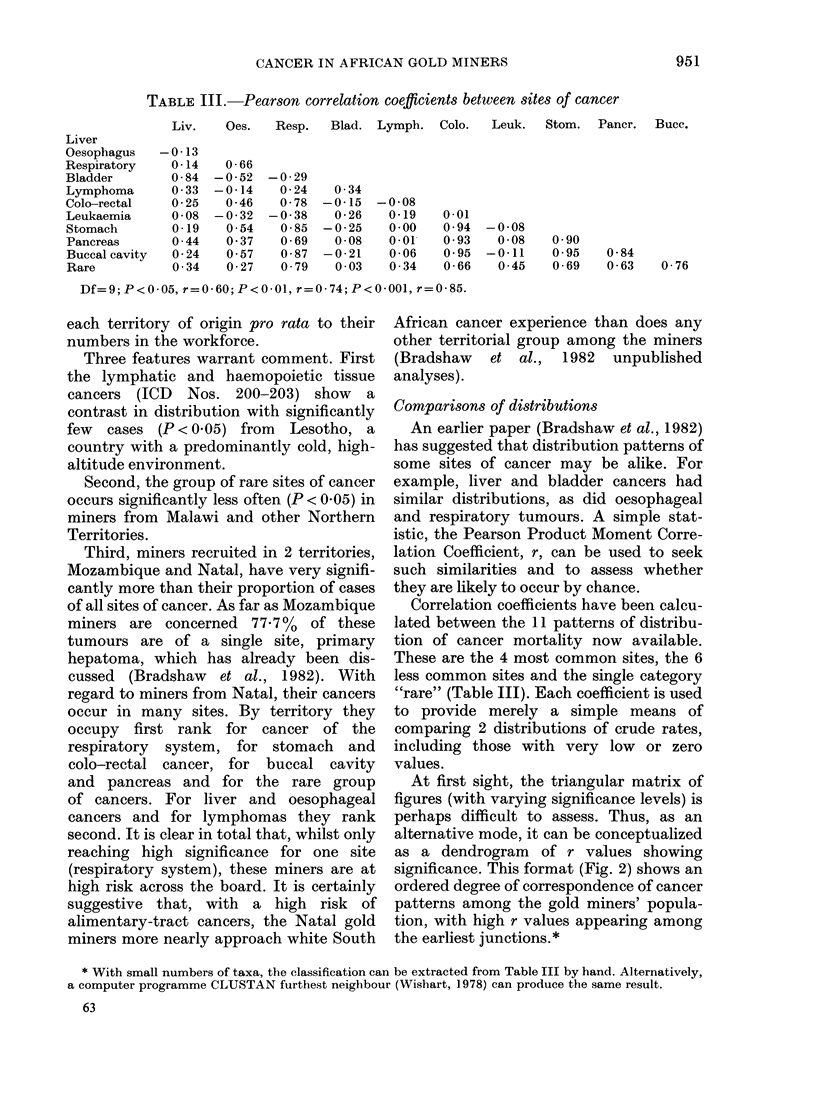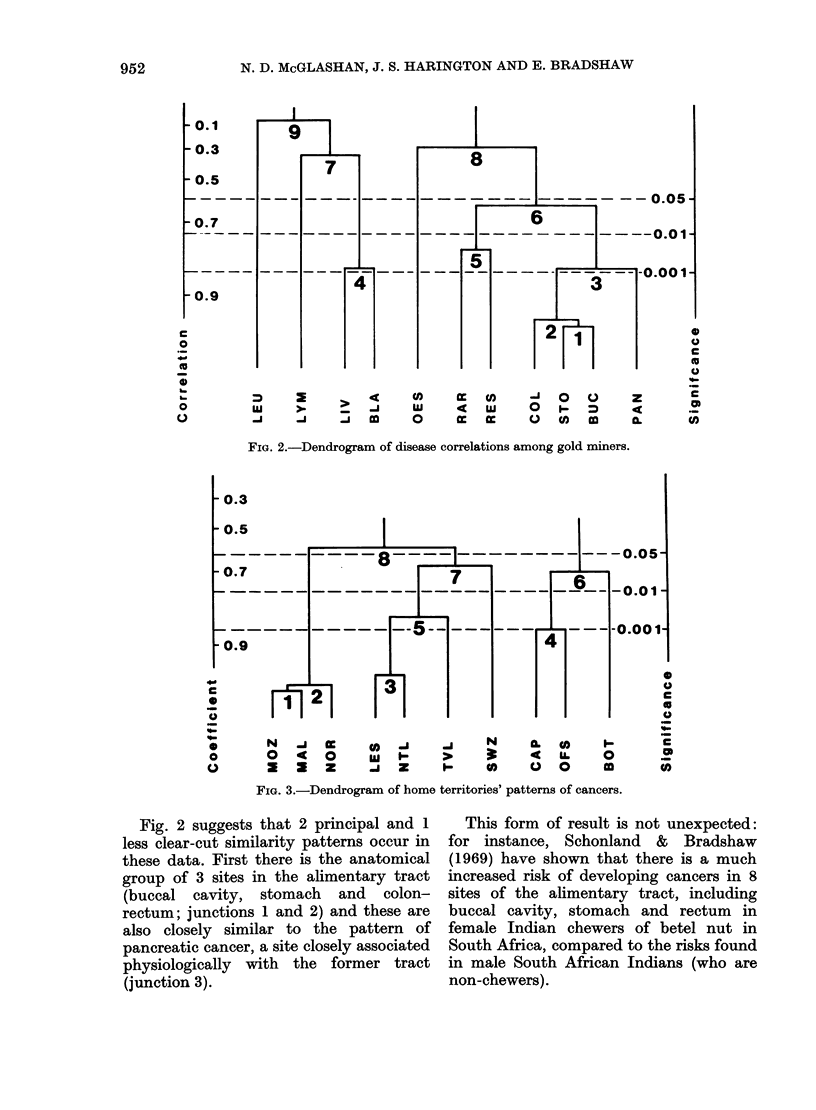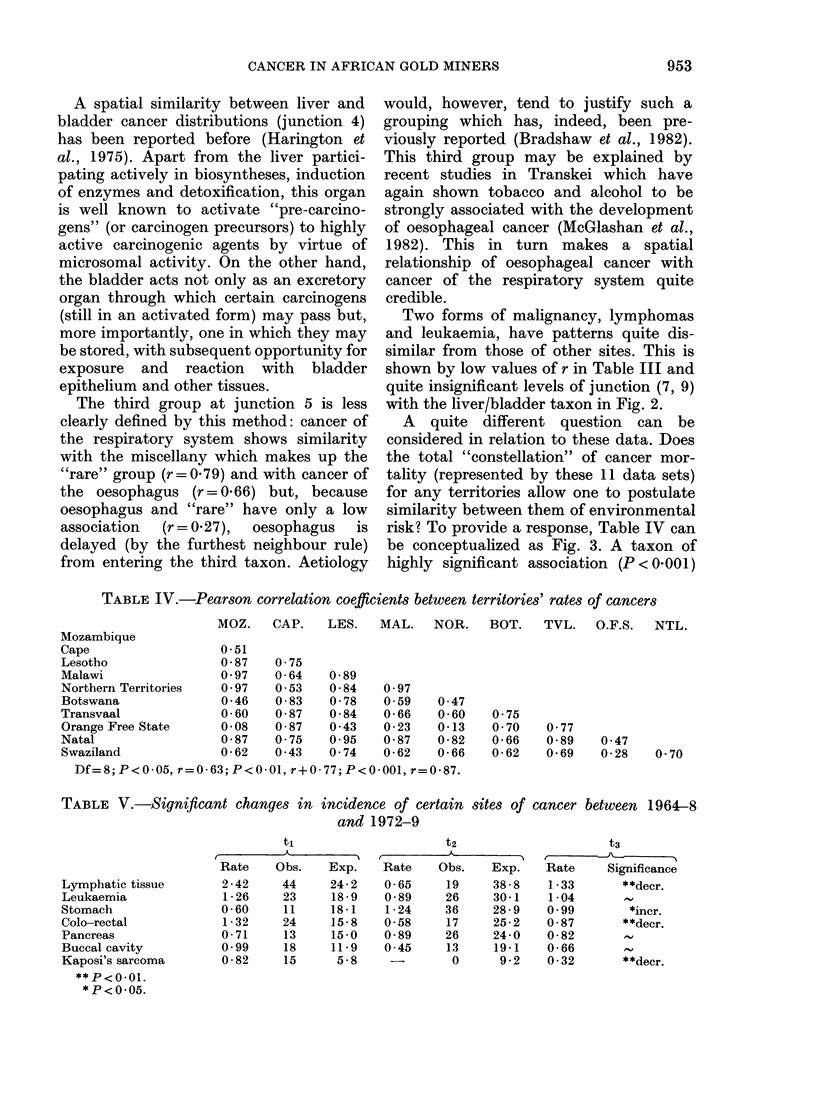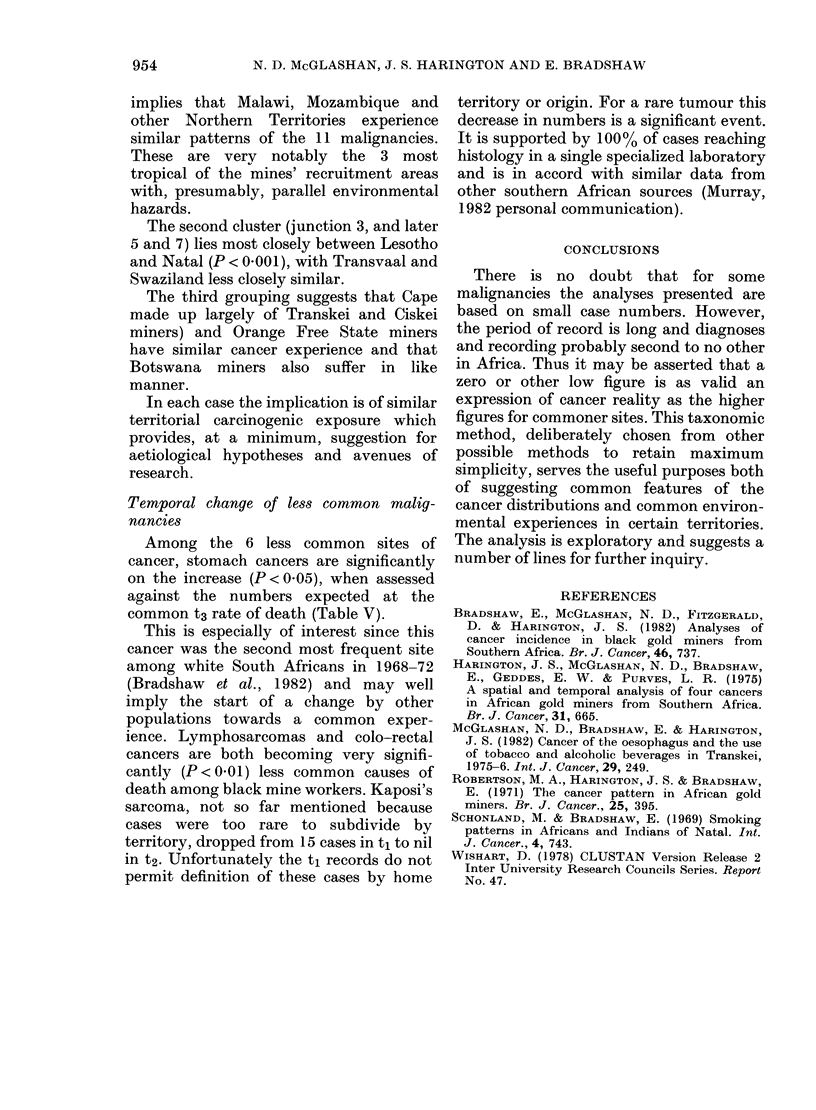# Eleven sites of cancer in black gold miners from Southern Africa: a geographic enquiry.

**DOI:** 10.1038/bjc.1982.306

**Published:** 1982-12

**Authors:** N. D. McGlashan, J. S. Harington, E. Bradshaw

## Abstract

The 5-year study of cancer in black gold miners, 1964-68, previously reported (Robertson et al., 1971) has now been extended for a separate 8-year period, 1972-79. This allows analyses of all cancers together and of 6 less common sites of cancer severally: lymphosarcomas, colon and rectum, leukaemia, stomach, pancreas and buccal cavity and also of those too rare to classify. The malignancies are considered by territory of origin of the gold miners. Lesotho miners have significantly fewer (P less than 0.05) tumours of the lymphatic and haemopoietic tissues and Natal miners have the highest incidence rates for 5 of the 6 sites (excluding leukaemia). A simple grouping method is applied to determine which of the 11 sites of cancer in the miners have similar distributions in their 10 territories of origin. The aetiological implications of clusters over space of certain sites of cancer are discussed. Finally, temporal change over the years 1964-79 shows a significant decrease overall (P less than 0.01) in cases of lymphosarcomas and colo-rectal cancers and an increase (P less than 0.05) in stomach cancer. The rare tumour, Kaposi's sarcoma, has also decreased significantly between the 2 periods studied.


*Br. J. Cancer* (1982) **46**, 947

### ELEVEN SITES OF CANCER IN BLACK GOLD MINERS FROM SOUTHERN AFRICA: A GEOGRAPHIC ENQUIRY

N. D. McGLASHAN, J. S. HARINGTON*† AND E. BRADSHAW*

*From the University of Tasmania, Hobart, Australia* 7001, *and*
**National Cancer Association of South Africa, Johannesburg*

Received 19 August 1981 Accepted 23 August 1982

**Summary.—The 5-year study of cancer in black gold miners, 1964–68, previously reported (Robertson *et al.*, 1971) has now been extended for a separate 8-year period, 1972–79. This allows analyses of all cancers together and of 6 less common sites of cancer severally: lymphosarcomas, colon and rectum, leukaemia, stomach, pancreas and buccal cavity and also of those too rare to classify. The malignancies are considered by territory of origin of the gold miners. Lesotho miners have significantly fewer ($P < 0{\cdot}05$) tumours of the lymphatic and haemopoietic tissues and Natal miners have the highest incidence rates for 5 of the 6 sites (excluding leukaemia). A simple grouping method is applied to determine which of the 11 sites of cancer in the miners have similar distributions in their 10 territories of origin. The aetiological implications of clusters over space of certain sites of cancer are discussed. Finally, temporal change over the years 1964–79 shows a significant decrease overall ($P < 0{\cdot}01$) in cases of lymphosarcomas and colo–rectal cancers and an increase ($P < 0{\cdot}05$) in stomach cancer. The rare tumour, Kaposi's sarcoma, has also decreased significantly between the 2 periods studied.**

ANALYSES published by Robertson *et al.*, (1971), Harington *et al.* (1975) and Bradshaw *et al.* (1982) have considered the spatial and temporal patterns of the 4 most common sites of cancer recorded among black gold miners recruited from homes in, respectively, 11 and 10 territories in 2 consecutive 8-year periods, 1964–1971 and 1972–1979 (see Fig. 1 for total gold-mine labour force from each home territory).

### METHODS

The methods of analysis and the limitations of the data-base, especially with regard to both age of recruitment and age of death, have been reported previously (Harington *et al.*, 1975). Briefly, it should be stressed that recruiting was of fully medically-examined men of apparent age between 18 and 40, but in the absence of birth-certificate records, actual age was neither known nor recorded by the mines of employment. Although an estimated age of each death was recorded, it was not possible to calculate age-specific mortality rates. We have previously suggested that the crude death rates were probably equivalent to an age-specific rate for the age group 25–35 years (Harington *et al.*, 1975). Diagnoses were made in the well equipped hospitals of the major mining groups, a high proportion being confirmed histologically in the laboratories of the South African Institute for Medical Research. The 4 most common sites (in rank order, primary liver, oesophagus, respiratory system and bladder) comprised 75·1% of all cancers in the earlier period and 79·1% in the later period.

The purpose of this paper is to examine the remaining data which refer to less common sites of cancer. These are sites of which, in an 8-year period, inadequate numbers of cases occurred for analysis but for which useful information emerges in a longer period of record. Unfortunately data are now extant

† Correspondence: Dr J. S. Harington.

FIG. 1.—Map of gold miners by territories of recruitment as percentage of all gold miners, 1964–68 and 1972–79 combined.

only for 5 years (1964–68 inclusive) of the earlier period, 1964–71. The former is referred to here as $t_1$. In it, the Ciskei and Transkei data have been combined to form one "Cape" group, thus reducing the number of areas to 10. The second period, $t_2$, refers to the 8 years 1972–79, and third, the accumulation of 13 years' data, made up of $t_1 + t_2$ is referred to as $t_3$.

### RESULTS AND DISCUSSION

### *Spatial analyses*

Patterns of distribution of the 4 most common sites of cancer among the black gold miners' labour force have already been described (Bradshaw *et al.*, 1982). The next 6 in rank order (with crude rates per 100,000 man-years) for the total time period, $t_3$, were, in 5th place, lymphosarcomas (1·33), then leukaemia (stomach), colo–rectal (0·87), pancreas (0·82) and, 10th, buccal cavity (0·66) (Table I).

In this 13-year period of $t_3$ a further 7·7% (142 cases) were of even less common sites. Since these individually remain too unusual to warrant separate analyses they have been grouped under the heading "rare" so that the total data-base of all cancers, 1807 cases in 13 years, is taken in full into these analyses (Table II). Among the 6 less common sites of cancer rates by territory vary from zero cases to 3·57 per 100,000 man-years and for the rare sites grouped together from 1·63 to 6·79.

As a test for the significance of these generally low case numbers the Poisson test has been employed, so that distinction can be drawn between small local variations and those for which explanation may be sought. In Table II, the actual cases recorded (observed) are compared with those that would be "expected" if each cancer were evenly distributed at the overall (total) rate among the miners from

TABLE I.—*Spatial variation of* 11 *sites of cancer and of all cancers among black gold miners by home territory* (1964–68 *and* 1972–79) (*crude rates per* 100,000 *man-years*)

| | Liver | Oeso-phagus | Respir-atory | Bladder | Lymph-oma | Leukaemia | Stomach | Colo-rectal | Pancreas | Buccal cavity | "Rare" | All cancers |
|---|---|---|---|---|---|---|---|---|---|---|---|---|
| Mozambique | 56·95 | 1·25 | 1·87 | 4·26 | 1·97 | 0·83 | 0·52 | 0·62 | 0·94 | 0·42 | 3·64 | 73·27 |
| Cape | 11·49 | 15·16 | 4·77 | 0·39 | 1·02 | 0·86 | 1·41 | 1·09 | 0·94 | 1·09 | 3·13 | 41·35 |
| Lesotho | 5·16 | 2·26 | 1·93 | 0·32 | 0·64 | 1·18 | 0·97 | 1·07 | 0·75 | 0·54 | 2·36 | 17·19 |
| Malawi | 9·66 | 2·63 | 0·88 | 1·93 | 1·05 | 1·76 | 0·70 | 0·70 | 1·05 | 0 | {1·63 | 22·92 |
| Northern Territories | 14·29 | 1·79 | 0·60 | 1·79 | 3·57 | 2·38 | 0 | 0 | 0 | 0 | | |
| Botswana | 2·66 | 3·04 | 1·14 | 0·76 | 0·38 | 1·14 | 1·14 | 0·76 | 0·38 | 0·76 | 2·66 | 14·83 |
| Transvaal | 9·27 | 7·72 | 7·72 | 1·02 | 2·06 | 0·51 | 1·03 | 1·03 | 0·51 | 1·03 | 4·12 | 36·03 |
| Orange Free State | 2·46 | 9·24 | 4·31 | 0 | 1·23 | 0 | 0·62 | 0 | 0 | 0 | 1·85 | 19·71 |
| Natal | 27·16 | 10·19 | 12·73 | 0·85 | 2·55 | 0·85 | 4·24 | 2·55 | 2·55 | 2·55 | 6·79 | 73·00 |
| Swaziland | 4·77 | 1·19 | 2·38 | 1·19 | 2·38 | 0 | 0 | 0 | 0 | 0 | 4·77 | 16·68 |
| Total | 18·75 | 6·25 | 3·07 | 1·46 | 1·33 | 1·04 | 0·99 | 0·87 | 0·82 | 0·66 | 3·00 | 38·19 |

TABLE II.—*Significance of case numbers of* 11 *sites of cancer and of all cancers among black gold miners by home territory* (1964–68 *and* 1972–79)

| | Populations (man-years) | Lymphatic and haemopoietic (tissue (200–203) | | Leukaemia (204–207) | | Stomach (151) | | Colo–rectal (152–154) | | Pancreas (157) | | Buccal cavity (140–149) | | Other malignancies | | Total all cancers | |
|---|---|---|---|---|---|---|---|---|---|---|---|---|---|---|---|---|---|
| | | Obs. | Exp. | Obs. | Exp. | Obs. | Exp. | Obs. | Exp. | Obs. | Exp. | Obs. | Exp. | Obs. | Exp. | Obs. | Exp. |
| Mozambique | 962236 | 19 | 12·8 | 8 | 10·0 | 5 | 9·6 | 6 | 8·3 | 9 | 7·9 | 4 | 6·3 | 35 | 28·9 | 705$^{++}$ | 367·5 |
| Cape | 1279412 | 13 | 17·0 | 11 | 13·3 | 18 | 12·7 | 14 | 11·1 | 12 | 10·5 | 14 | 8·4 | 40 | 38·4 | 529 | 488·6 |
| Lesotho | 930689 | 6$^{-}$ | 12·4 | 11 | 9·6 | 9 | 9·2 | 10 | 8·1 | 7 | 7·7 | 5 | 6·1 | 22 | 27·9 | 160$^{--}$ | 355·5 |
| Malawi | 569525 | 6 | 7·6 | 10 | 5·9 | 4 | 5·7 | 4 | 4·9 | 6 | 4·7 | 0 | 3·7 | 12$^{-}$ | 22·1 | 169$^{--}$ | 281·7 |
| Northern Territories | 167978 | 6 | 2·2 | 4 | 1·7 | 0 | 1·7 | 0 | 1·5 | 0 | 1·4 | 0 | 1·1 | | | | |
| Botswana | 263035 | 1 | 3·5 | 3 | 2·7 | 3 | 2·6 | 2 | 2·3 | 1 | 2·2 | 2 | 1·7 | 7 | 7·9 | 39$^{--}$ | 100·5 |
| Transvaal | 194272 | 4 | 2·6 | 1 | 2·0 | 2 | 1·9 | 2 | 1·7 | 1 | 1·6 | 2 | 1·3 | 8 | 5·8 | 70 | 74·2 |
| Orange Free State | 162394 | 2 | 2·2 | 0 | 1·7 | 1 | 1·6 | 0 | 1·4 | 0 | 1·3 | 0 | 1·1 | 3 | 4·9 | 32$^{--}$ | 62·0 |
| Natal | 117811 | 3 | 1·6 | 1 | 1·2 | 5 | 1·2 | 3 | 1·0 | 3 | 1·0 | 3 | 0·8 | 8 | 3·5 | 86$^{++}$ | 45·0 |
| Swaziland | 83932 | 2 | 1·1 | 0 | 0·9 | 0 | 0·8 | 0 | 0·7 | 0 | 0·7 | 0 | 0·5 | 4 | 2·5 | 14$^{--}$ | 32·1 |
| Total | 4731284 | 63* | | 49 | | 47 | | 41 | | 39 | | 31* | | 142† | | 1807† | |

$P<0·05^{-}$ (Obs < Exp); $P<0·05^{+}$ (Obs > Exp); $P<0·01^{--}$ (Obs < Exp); $P<0·01^{++}$ (Obs > Exp).
* includes one case of unknown home origin.
† includes 3 cases of unknown home origin.

TABLE III.—*Pearson correlation coefficients between sites of cancer*

| | Liv. | Oes. | Resp. | Blad. | Lymph. | Colo. | Leuk. | Stom. | Pancr. | Bucc. |
|---|---|---|---|---|---|---|---|---|---|---|
| Liver | | | | | | | | | | |
| Oesophagus | −0·13 | | | | | | | | | |
| Respiratory | 0·14 | 0·66 | | | | | | | | |
| Bladder | 0·84 | −0·52 | −0·29 | | | | | | | |
| Lymphoma | 0·33 | −0·14 | 0·24 | 0·34 | | | | | | |
| Colo–rectal | 0·25 | 0·46 | 0·78 | −0·15 | −0·08 | | | | | |
| Leukaemia | 0·08 | −0·32 | −0·38 | 0·26 | 0·19 | 0·01 | | | | |
| Stomach | 0·19 | 0·54 | 0·85 | −0·25 | 0·00 | 0·94 | −0·08 | | | |
| Pancreas | 0·44 | 0·37 | 0·69 | 0·08 | 0·01 | 0·93 | 0·08 | 0·90 | | |
| Buccal cavity | 0·24 | 0·57 | 0·87 | −0·21 | 0·06 | 0·95 | −0·11 | 0·95 | 0·84 | |
| Rare | 0·34 | 0·27 | 0·79 | 0·03 | 0·34 | 0·66 | 0·45 | 0·69 | 0·63 | 0·76 |

Df = 9; $P < 0{\cdot}05$, $r = 0{\cdot}60$; $P < 0{\cdot}01$, $r = 0{\cdot}74$; $P < 0{\cdot}001$, $r = 0{\cdot}85$.

each territory of origin *pro rata* to their numbers in the workforce.

Three features warrant comment. First the lymphatic and haemopoietic tissue cancers (ICD Nos. 200–203) show a contrast in distribution with significantly few cases ($P < 0{\cdot}05$) from Lesotho, a country with a predominantly cold, high-altitude environment.

Second, the group of rare sites of cancer occurs significantly less often ($P < 0{\cdot}05$) in miners from Malawi and other Northern Territories.

Third, miners recruited in 2 territories, Mozambique and Natal, have very significantly more than their proportion of cases of all sites of cancer. As far as Mozambique miners are concerned 77·7% of these tumours are of a single site, primary hepatoma, which has already been discussed (Bradshaw *et al.*, 1982). With regard to miners from Natal, their cancers occur in many sites. By territory they occupy first rank for cancer of the respiratory system, for stomach and colo–rectal cancer, for buccal cavity and pancreas and for the rare group of cancers. For liver and oesophageal cancers and for lymphomas they rank second. It is clear in total that, whilst only reaching high significance for one site (respiratory system), these miners are at high risk across the board. It is certainly suggestive that, with a high risk of alimentary-tract cancers, the Natal gold miners more nearly approach white South African cancer experience than does any other territorial group among the miners (Bradshaw *et al.*, 1982 unpublished analyses).

*Comparisons of distributions*

An earlier paper (Bradshaw *et al.*, 1982) has suggested that distribution patterns of some sites of cancer may be alike. For example, liver and bladder cancers had similar distributions, as did oesophageal and respiratory tumours. A simple statistic, the Pearson Product Moment Correlation Coefficient, $r$, can be used to seek such similarities and to assess whether they are likely to occur by chance.

Correlation coefficients have been calculated between the 11 patterns of distribution of cancer mortality now available. These are the 4 most common sites, the 6 less common sites and the single category "rare" (Table III). Each coefficient is used to provide merely a simple means of comparing 2 distributions of crude rates, including those with very low or zero values.

At first sight, the triangular matrix of figures (with varying significance levels) is perhaps difficult to assess. Thus, as an alternative mode, it can be conceptualized as a dendrogram of $r$ values showing significance. This format (Fig. 2) shows an ordered degree of correspondence of cancer patterns among the gold miners' population, with high $r$ values appearing among the earliest junctions.*

* With small numbers of taxa, the classification can be extracted from Table III by hand. Alternatively, a computer programme CLUSTAN furthest neighbour (Wishart, 1978) can produce the same result.

Fig. 2.—Dendrogram of disease correlations among gold miners.

Fig. 3.—Dendrogram of home territories' patterns of cancers.

Fig. 2 suggests that 2 principal and 1 less clear-cut similarity patterns occur in these data. First there is the anatomical group of 3 sites in the alimentary tract (buccal cavity, stomach and colon–rectum; junctions 1 and 2) and these are also closely similar to the pattern of pancreatic cancer, a site closely associated physiologically with the former tract (junction 3).

This form of result is not unexpected: for instance, Schonland & Bradshaw (1969) have shown that there is a much increased risk of developing cancers in 8 sites of the alimentary tract, including buccal cavity, stomach and rectum in female Indian chewers of betel nut in South Africa, compared to the risks found in male South African Indians (who are non-chewers).

A spatial similarity between liver and bladder cancer distributions (junction 4) has been reported before (Harington *et al.*, 1975). Apart from the liver participating actively in biosyntheses, induction of enzymes and detoxification, this organ is well known to activate "pre-carcinogens" (or carcinogen precursors) to highly active carcinogenic agents by virtue of microsomal activity. On the other hand, the bladder acts not only as an excretory organ through which certain carcinogens (still in an activated form) may pass but, more importantly, one in which they may be stored, with subsequent opportunity for exposure and reaction with bladder epithelium and other tissues.

The third group at junction 5 is less clearly defined by this method: cancer of the respiratory system shows similarity with the miscellany which makes up the "rare" group ($r = 0{\cdot}79$) and with cancer of the oesophagus ($r = 0{\cdot}66$) but, because oesophagus and "rare" have only a low association ($r = 0{\cdot}27$), oesophagus is delayed (by the furthest neighbour rule) from entering the third taxon. Aetiology would, however, tend to justify such a grouping which has, indeed, been previously reported (Bradshaw *et al.*, 1982). This third group may be explained by recent studies in Transkei which have again shown tobacco and alcohol to be strongly associated with the development of oesophageal cancer (McGlashan *et al.*, 1982). This in turn makes a spatial relationship of oesophageal cancer with cancer of the respiratory system quite credible.

Two forms of malignancy, lymphomas and leukaemia, have patterns quite dissimilar from those of other sites. This is shown by low values of $r$ in Table III and quite insignificant levels of junction (7, 9) with the liver/bladder taxon in Fig. 2.

A quite different question can be considered in relation to these data. Does the total "constellation" of cancer mortality (represented by these 11 data sets) for any territories allow one to postulate similarity between them of environmental risk? To provide a response, Table IV can be conceptualized as Fig. 3. A taxon of highly significant association ($P < 0{\cdot}001$)

TABLE IV.—*Pearson correlation coefficients between territories' rates of cancers*

| | MOZ. | CAP. | LES. | MAL. | NOR. | BOT. | TVL. | O.F.S. | NTL. |
|---|---|---|---|---|---|---|---|---|---|
| Mozambique | | | | | | | | | |
| Cape | 0·51 | | | | | | | | |
| Lesotho | 0·87 | 0·75 | | | | | | | |
| Malawi | 0·97 | 0·64 | 0·89 | | | | | | |
| Northern Territories | 0·97 | 0·53 | 0·84 | 0·97 | | | | | |
| Botswana | 0·46 | 0·83 | 0·78 | 0·59 | 0·47 | | | | |
| Transvaal | 0·60 | 0·87 | 0·84 | 0·66 | 0·60 | 0·75 | | | |
| Orange Free State | 0·08 | 0·87 | 0·43 | 0·23 | 0·13 | 0·70 | 0·77 | | |
| Natal | 0·87 | 0·75 | 0·95 | 0·87 | 0·82 | 0·66 | 0·89 | 0·47 | |
| Swaziland | 0·62 | 0·43 | 0·74 | 0·62 | 0·66 | 0·62 | 0·69 | 0·28 | 0·70 |

Df=8; $P < 0{\cdot}05$, $r = 0{\cdot}63$; $P < 0{\cdot}01$, $r + 0{\cdot}77$; $P < 0{\cdot}001$, $r = 0{\cdot}87$.

TABLE V.—*Significant changes in incidence of certain sites of cancer between* 1964–8 *and* 1972–9

| | $t_1$ | | | $t_2$ | | | $t_3$ | |
|---|---|---|---|---|---|---|---|---|
| | Rate | Obs. | Exp. | Rate | Obs. | Exp. | Rate | Significance |
| Lymphatic tissue | 2·42 | 44 | 24·2 | 0·65 | 19 | 38·8 | 1·33 | **decr. |
| Leukaemia | 1·26 | 23 | 18·9 | 0·89 | 26 | 30·1 | 1·04 | ~ |
| Stomach | 0·60 | 11 | 18·1 | 1·24 | 36 | 28·9 | 0·99 | *incr. |
| Colo–rectal | 1·32 | 24 | 15·8 | 0·58 | 17 | 25·2 | 0·87 | **decr. |
| Pancreas | 0·71 | 13 | 15·0 | 0·89 | 26 | 24·0 | 0·82 | ~ |
| Buccal cavity | 0·99 | 18 | 11·9 | 0·45 | 13 | 19·1 | 0·66 | ~ |
| Kaposi's sarcoma | 0·82 | 15 | 5·8 | — | 0 | 9·2 | 0·32 | **decr. |

** $P < 0{\cdot}01$.
* $P < 0{\cdot}05$.

implies that Malawi, Mozambique and other Northern Territories experience similar patterns of the 11 malignancies. These are very notably the 3 most tropical of the mines' recruitment areas with, presumably, parallel environmental hazards.

The second cluster (junction 3, and later 5 and 7) lies most closely between Lesotho and Natal ($P < 0{\cdot}001$), with Transvaal and Swaziland less closely similar.

The third grouping suggests that Cape made up largely of Transkei and Ciskei miners) and Orange Free State miners have similar cancer experience and that Botswana miners also suffer in like manner.

In each case the implication is of similar territorial carcinogenic exposure which provides, at a minimum, suggestion for aetiological hypotheses and avenues of research.

*Temporal change of less common malignancies*

Among the 6 less common sites of cancer, stomach cancers are significantly on the increase ($P < 0{\cdot}05$), when assessed against the numbers expected at the common $t_3$ rate of death (Table V).

This is especially of interest since this cancer was the second most frequent site among white South Africans in 1968–72 (Bradshaw *et al.*, 1982) and may well imply the start of a change by other populations towards a common experience. Lymphosarcomas and colo-rectal cancers are both becoming very significantly ($P < 0{\cdot}01$) less common causes of death among black mine workers. Kaposi's sarcoma, not so far mentioned because cases were too rare to subdivide by territory, dropped from 15 cases in $t_1$ to nil in $t_2$. Unfortunately the $t_1$ records do not permit definition of these cases by home territory or origin. For a rare tumour this decrease in numbers is a significant event. It is supported by 100% of cases reaching histology in a single specialized laboratory and is in accord with similar data from other southern African sources (Murray, 1982 personal communication).

### CONCLUSIONS

There is no doubt that for some malignancies the analyses presented are based on small case numbers. However, the period of record is long and diagnoses and recording probably second to no other in Africa. Thus it may be asserted that a zero or other low figure is as valid an expression of cancer reality as the higher figures for commoner sites. This taxonomic method, deliberately chosen from other possible methods to retain maximum simplicity, serves the useful purposes both of suggesting common features of the cancer distributions and common environmental experiences in certain territories. The analysis is exploratory and suggests a number of lines for further inquiry.

### REFERENCES

BRADSHAW, E., McGLASHAN, N. D., FITZGERALD, D. & HARINGTON, J. S. (1982) Analyses of cancer incidence in black gold miners from Southern Africa. *Br. J. Cancer*, **46**, 737.

HARINGTON, J. S., McGLASHAN, N. D., BRADSHAW, E., GEDDES, E. W. & PURVES, L. R. (1975) A spatial and temporal analysis of four cancers in African gold miners from Southern Africa. *Br. J. Cancer*, **31**, 665.

McGLASHAN, N. D., BRADSHAW, E. & HARINGTON, J. S. (1982) Cancer of the oesophagus and the use of tobacco and alcoholic beverages in Transkei, 1975–6. *Int. J. Cancer*, **29**, 249.

ROBERTSON, M. A., HARINGTON, J. S. & BRADSHAW, E. (1971) The cancer pattern in African gold miners. *Br. J. Cancer.*, **25**, 395.

SCHONLAND, M. & BRADSHAW, E. (1969) Smoking patterns in Africans and Indians of Natal. *Int. J. Cancer.*, **4**, 743.

WISHART, D. (1978) CLUSTAN Version Release 2 Inter University Research Councils Series. *Report* No. 47.